# Intelligent Temperature-Control of Drilling Fluid in Natural Gas Hydrate Formation by Nano-Silica/Modified n-Alkane Microcapsules

**DOI:** 10.3390/nano11092370

**Published:** 2021-09-12

**Authors:** Yubin Zhang, Zhengsong Qiu, Jiaxing Mu, Yongle Ma, Xin Zhao, Hanyi Zhong, Weian Huang, Pengfei Guo

**Affiliations:** 1 School of Petroleum Engineering, China University of Petroleum (East China), Qingdao 266580, China; upczhangyubin@126.com (Y.Z.); zhgw1027@163.com (J.M.); upczhao123@163.com (X.Z.); upczhong1112@163.com (H.Z.); upcschooler@163.com (W.H.); upcbron0912@163.com (P.G.); 2 Drilling Technology Research and Development Center, Bo-Xing Division of CNPC Offshore Engineering Company Limited, Tianjin 300451, China; 15853291860@163.com

**Keywords:** microcapsule, modified n-alkane, nano-silica, hydrate formation, latent heat performance, intelligent temperature-control

## Abstract

Inhibiting hydrate decomposition due to the friction heat generated by the drilling tools is one of the key factors for drilling hydrate formation. Since the existing method based on chemical inhibition technology can only delay the hydrate decomposition rate, a phase-change microcapsule was introduced in this paper to inhibit, by the intelligent control of the drilling fluid temperature, the decomposition of the formation hydrate, which was microencapsulated by modified n-alkane as the core material, and nano-silica was taken as the shell material. Scanning electron microscope (SEM), size distribution, X-ray diffraction (XRD), and Fourier transform infrared spectrometer (FT-IR) were utilized to characterize the structural properties of microcapsules. Differential scanning calorimetry (DSC) spectra displayed that the latent heat was 136.8 J/g in the case of melting enthalpy and 136.4 J/g in the case of solidification enthalpy, with an encapsulation efficiency of 62.6%. In addition, the prepared microcapsules also showed good thermal conductivity and reliability. By comparison, it was also proved that the microcapsules had good compatibility with drilling fluid, which can effectively control the temperature of drilling fluid for the inhibition of hydrate decomposition.

## 1. Introduction

The huge resource reserves of natural gas hydrate have made the drilling and development of hydrate formation a research hotspot [1,2]. Hydrate mainly occurs under low temperature and high pressure in seabed formation, which puts forward a severe technical challenge to drilling engineering [3,4]. One of the key problems lies in the hydrate decomposition caused by the cutting heat of drilling tools at the long horizontal well section in hydrate formation, which causes the formation to lose skeleton support, leading to collapse and even geological disasters [5,6,7]. However, the existing technology only delays the hydrate decomposition rate to a certain extent by the addition of chemical treatment agent [8,9] instead of changing the phase equilibrium temperature of hydrate, therefore, the formation hydrate will continuously decompose with the increase of temperature. In that case, the intelligent temperature-control of drilling fluid by phase change materials (PCMs) to inhibit hydrate decomposition is of significant research value.

In recent years, scholars have carried out a large amount of researches on PCMs, and the technology of temperature control has also been developed gradually by storing energy in advance through material phase transition and releasing it at a suitable temperature [10,11,12,13]. These PCMs are mainly divided into organic and inorganic compounds. Compared with organic PCMs, inorganic PCMs are usually characterized by lower cost, higher thermal conductivity, and subcooling [14,15]. However, organic PCMs has been more widely used because of their higher latent heat performance, and n-alkanes are one of the effective ones [16,17,18]. For example, polystyrene, melamine formaldehyde, and capric-stearic acid have been used to coat n-tetradecane and n-hexadecane [19,20,21]. Compared with these shell materials, nano-silica can better improve the thermal conductivity, showing good hydrophilicity [22,23]. Therefore, this paper proposed a new idea of inhibiting the decomposition of formation hydrate by phase change microcapsules, which were synthesized by interfacial polymerization using modified n-alkane as the core material and nano-silica formed by simultaneous hydrolysis and polycondensation of TEOS and APTS as the shell material.

**Figure 1:**
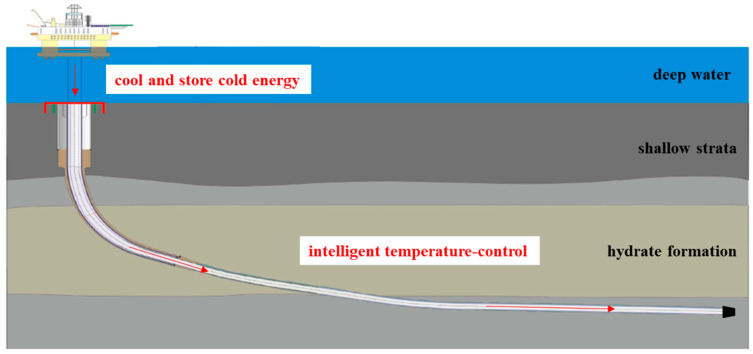
Schematic diagram of microcapsules intelligent temperature-control in hydrate formation (modified from [25]).

The water depth in the vertical well section of hydrate formation exceeds 1000 m, and the seabed temperature ranges from 2–4 °C [24]. As shown in Figure 1, microcapsules change phase to store cold energy by cooling from wellhead to seabed. When circulating to the horizontal well section, which contains the hydrate in the formation, the microcapsules release the cold energy to balance the heat generated during the drilling process to control the temperature of the drilling fluid, so as to inhibit the decomposition of formation hydrate. In this paper, the structural properties of microcapsules were characterized by SEM, size distribution, XRD, and FT-IR tests. The latent heat of microcapsules was obtained by measuring DSC spectra, and then the encapsulation efficiency was calculated. After that, the thermal conductivity and reliability of microcapsules were discussed. Moreover, to better simulate the field application effect, the thermoregulating performance and compatibility between microcapsules and drilling fluid were experimentally studied.

## 2. Methodology

### 2.1. Materials

Tetraethyl nano-silicate (TEOS) and 3-aminopropyl-triethoxysilane (APTS) were purchased from Aladdin Reagents (Shanghai, China). And N-tetradecane, n-hexadecane, fatty acid ester, Span 80, Tween 80, and ethanol were obtained from Sinopharm Chemicals (Beijing, China). In addition, nucleating agent SD-N and cementing agent SD-C were provided by Shida Chuangxin Technology Co., Ltd. (Dongying, China). Deionized water was made in the laboratory.

### 2.2. Preparation of Nano-Silica/Modified n-Alkane Microcapsules

**Figure 2:**
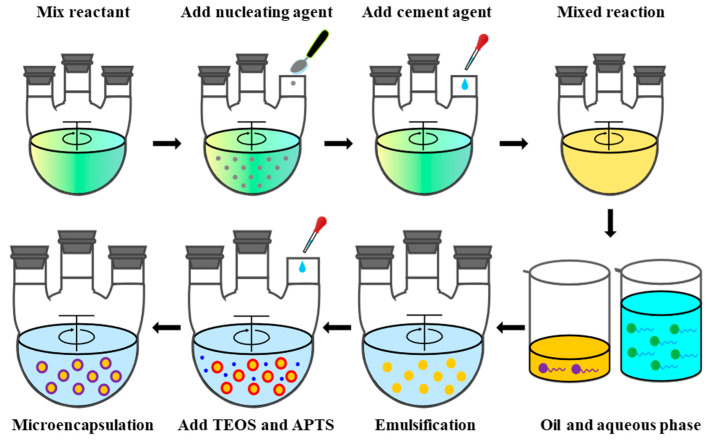
Typical preparation procedure of nano-silica/modified n-alkane microcapsules.

Microcapsules were prepared by interfacial polymerization, of which modified n-alkane was used as the core material, and nano-silica formed by simultaneous hydrolysis and polycondensation of TEOS and APTS was used as the shell material. In a typical preparation procedure (as shown in Figure 2), 10 g n-tetradecane, 25 g n-hexadecane, and 10 g fatty acid ester were mixed in a 100 mL three-necks flask, then the mixture was added with 0.68 g nucleating agent SD-N and stirred at 40 °C for 4.5 h. After that, with the increase of the temperature, 1.35 g cementing agent SD-C was added gradually. Then, the modified n-alkane was obtained by continuous stirring for 7 h, and 10 g modified n-alkane and 1.2 g Span 80 were mixed in a beaker as the oil phase. The aqueous phase was obtained by mixing 100 g deionized water with 1.5 g Tween 80. In a 250 mL three-necks flask, the oil phase and the aqueous phase were mixed and stirred (500 rpm) at 55 °C for the preparation of a stable oil/water (O/W) emulsion. Subsequently, 9.6 g TEOS was added slowly to the O/W emulsion, which was followed by the addition of 2.4 g APTS to adjust the pH value. After stirring for 5 h, the products were washed three times with ethanol before being dried at 45 °C for 16 h.

### 2.3. Experimental Methods

The microcapsules were performed at an accelerating voltage of 5 kV by using a scanning electron microscope (SEM. Zeiss Sigma 300) after being coated with gold. Transmission electron microscope (TEM) photos were obtained by a JEOL JEM 2100 equipment after ultrasonic dispersion in deionized water. A Bettersize 2000 laser particle sizer was used for the determination of the size distribution. The microcapsules were dispersed in deionized water by ultrasound and added to the test dish drop by drop until the shading rate reached 10%. X-ray diffraction (XRD) patterns were observed by using an Ultima IV equipment with the radiation voltage of 45 kV and the current of 40 mA at a rate of 2°/min within the range of 10–80°. Fourier transform infrared spectrometer (FT-IR, Thermo Scientific Nicolet 10) was used for the observation of the structural characteristics of microcapsules within the range of 4000–400 cm^−1^. Differential scanning calorimetry (DSC) spectra was measured by using a TA Q2000 instrument. An approximately 5 mg sample was used under nitrogen atmosphere at the heating rate ranging from −50 °C to 50 °C. The thermal reliability of microcapsules was determined by DSC results after thermal cycling experiment. The microcapsules were put into an RTP incubator for heating/cooling in the range of −50–50 ℃, which cycled for 10, 20, and 30 times, respectively.

A thermal conductivity measuring apparatus (TC3000E) was used for the measurement of the thermal conductivity of the microcapsules. The modified n-alkane was directly added to the sample cell and measured under liquid test mode. After being pressed into two cylinders, the powder microcapsules were tested by clamping the sensing probe between the smooth surfaces. At least three experiments were performed in each group to ensure parallelism. The thermoregulating performance evaluation experiment was implemented in a reactor equipped with a hotplate stirrer. The microcapsule solution was added to the reactor, and then the hotplate stirrer was operated at the stirring speed of 200 r/min and the power of 120 W to simulate the release of the cutting heat of drilling tools. The temperature of the microcapsule solution was continuously recorded to characterize the thermoregulating performance. The rheology was tested by using six-speed viscometer (SRV11), and the filtration was measured by medium pressure filtration meter (SD4B). According to the standard of American Petroleum Institute (API), apparent viscosity (AV), plastic viscosity (PV), and yield point (YP) were calculated by the following formulas [26,27], respectively:

AV =φ600/2 (mPa·s)(1)

PV = φ600 − φ300 (mPa·s)(2)

YP = (φ300 − PV)/2 (Pa)(3)

where φ600 and φ300 refer to the 600 and 300 rpm readings of the six-speed viscometer.

## 3. Results and Discussion

### 3.1. Synthesis of the Nano-Silica/Modified n-Alkane Microcapsules

**Figure 3:**
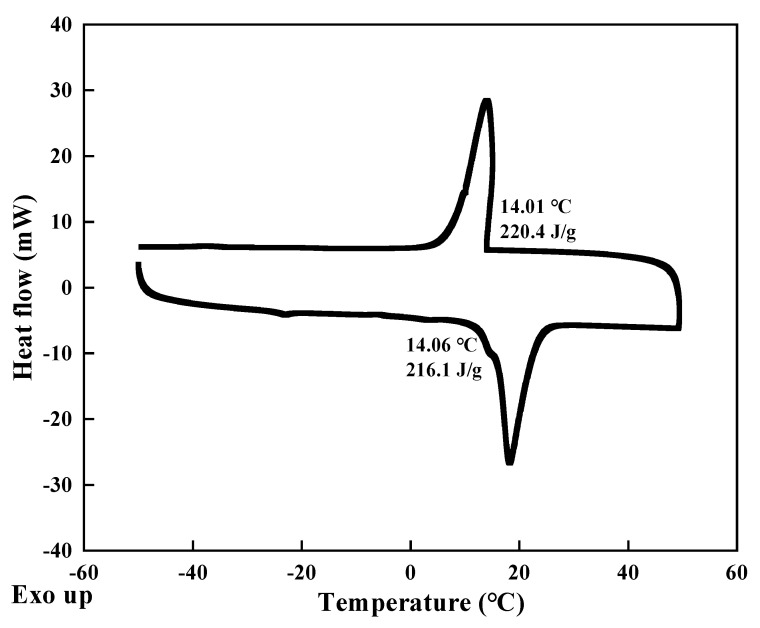
DSC spectra of prepared modified n-alkane.

The modified n-alkane PCM with a suitable phase-change temperature was prepared to meet the temperature requirement of hydrate formation in Shenhu block, South China Sea [24]. The DSC spectra is shown in Figure 3. The melting temperature is 14.06 °C with a melting enthalpy of 216.1 J/g, and the solidification temperature is 14.01 °C with a solidification enthalpy of 220.4 J/g. Microcapsules were synthesized by interfacial polymerization, of which modified n-alkane was used as the core material, and nano-silica formed by simultaneous hydrolysis and polycondensation of TEOS and APTS was used as the shell material. As shown in Figure 2, after the preparation of modified n-alkanes, deionized water and emulsifier were added to form a stable oil-in-water (O/W) emulsion. APTS was used together with TEOS to adjust the pH value of the emulsion system through their inherent [-NH_2_] groups, which release OH^-^, thereby facilitating the nucleophilic substitution and hydrolysis reaction of TEOS. Then, the polycondensation between hydrolysates Si-OH produced SiO_2_, and nano-silica shell was formed by the continuous deposition and growth of SiO_2_ at the O/W interface.

Latent heat performance is the key factor of PCMs, and encapsulation rate (R) and encapsulation efficiency (E) were used to characterize the latent heat properties of microcapsules prepared under different synthesis conditions, which can be expressed as follows [28,29]:

(4) $$R=\frac{\Delta \mathrm{Hm},\;\mathrm{Microcapsule}}{\Delta \mathrm{Hm},\mathrm{Modified}\;n-\mathrm{alkane}}$$

(5) $$E=\frac{\Delta \mathrm{Hm},\mathrm{Microcapsule}+\Delta H,\mathrm{Microcapsule}}{\Delta \mathrm{Hm},\mathrm{Modified}\;n-\mathrm{alkane}+\Delta \mathrm{Hs},\mathrm{Modified}\;n-\mathrm{alkane}}$$

where ∆Hm, Microcapsule and ∆Hm, Modified n-alkane refer to the melting enthalpy of the microcapsule and modified n-alkane, respectively; and ∆Hs, Microcapsule and ∆Hs, Modified n-alkane represent the solidification enthalpy of the microcapsule and modified n-alkane, respectively.

Table 1 shows the latent heat properties of microcapsules under different conditions. Encapsulation rate and efficiency of microcapsules were used to optimize the preparation conditions. The ratio of oil/water has an impact on the stability of the emulsion and the sphericity of the microcapsule. Neither too large nor too small ratio is conducive to achieving the oil–water balance and forming a stable O/W emulsion. The introduction of emulsifier is available for a significant reduction of the surface tension of the oil–water interface. However, the emulsion droplets formed were easy to break under low dosage, and the subsequent SiO_2_ deposition would be blocked under high dosage; both phenomena restricted the effective coating of nano-silica shell. A slight increase in the ratio of Tween 80 may result in the increase of the Hydrophile Lipophilic Balance (HLB) value, which makes the droplets more stable in O/W emulsion [30]. In the case that the core/shell ratio is large, nano-silica produced by hydrolysis and polycondensation will not be sufficient to completely cover the core material, resulting in the breakage of some microcapsules; while small core/shell ratio may lead to a shell that is too thick. Based on the above results, the latent heat of the nano-silica/modified n-alkane microcapsule is 136.8 (±2.0) J/g in the case of melting enthalpy and 136.4 (±1.8) J/g in the case of solidification enthalpy. It was calculated that the encapsulation rate and encapsulation efficiency were 63.3 (±0.9) % and 62.6 (±0.8) %, respectively. The comparison of encapsulation efficiency of different PCMs is displayed in Table 2, and the results showed that the nano-silica/modified n-alkane microcapsules prepared in this paper showed high latent heat and encapsulation efficiency, proving a good thermal energy storage capacity.

### 3.2. Structural Properties

**Figure 4:**
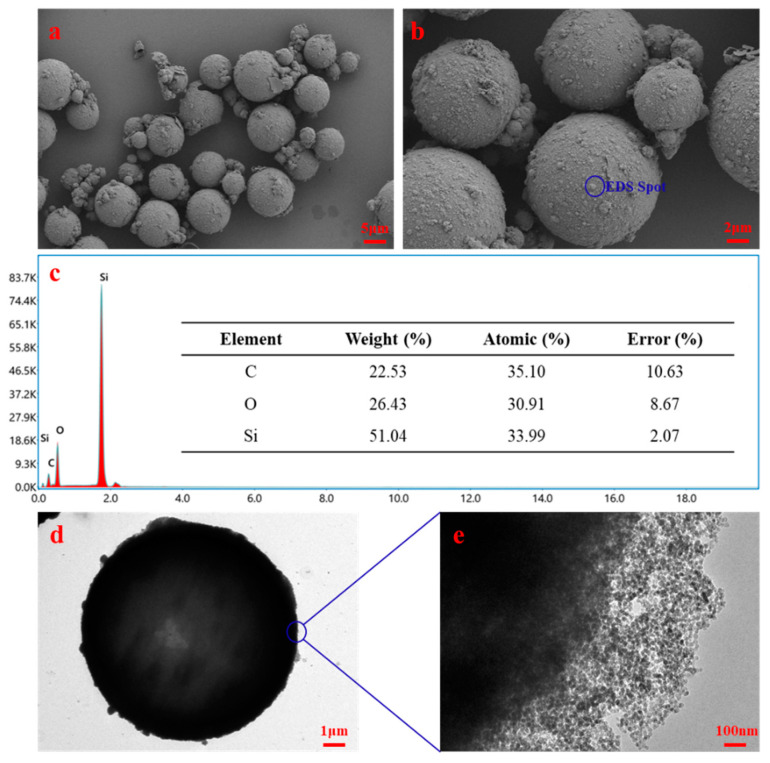
SEM photos (**a**,**b**), EDS spectrum (**c**), and TEM photos (**d**,**e**) of microcapsules.

The morphology of microcapsules was observed by using SEM and TEM, as shown in Figure 4. The synthesized microcapsules were in spherical shape with dense surface, most of which are in the particle size of 10 μm. As shown in the energy dispersion spectroscopy (EDS) spectrum in Figure 4c, since the core material contains no silicon element, the silicon on the surface of the microcapsules comes from nano-silica, indicating the successful coating. The results of TEM (as shown in Figure 4d) also proved that the microcapsules had good sphericity. Meanwhile, nano-silica was observed at the shell, which also indicated that the core material had been coated with nano-silica.

**Figure 5:**
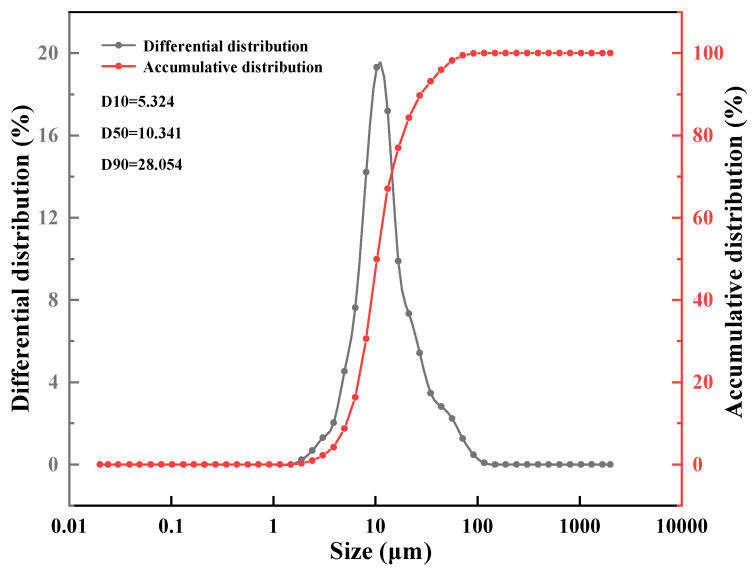
Particle size distribution of microcapsules in deionized water.

Figure 5 displays the particle size distribution of microcapsules in deionized water, and the particle size distribution curve showed a single peak, which indicated that the microcapsule size was relatively uniform. The microcapsules with median particle size was 10.341 μm; most of them were distributed within the range of 5.324–28.054 μm, which is consistent with the SEM test results. Meanwhile, the particle size also meets the screen requirements (106 μm) for offshore drilling recovery.

**Figure 6:**
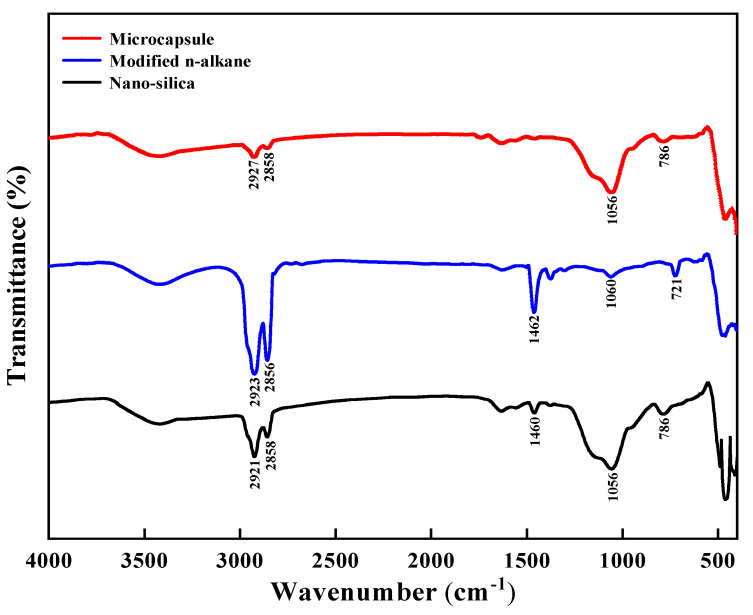
FTIR spectra of nano-silica, modified n-alkane and microcapsule.

**Figure 7:**
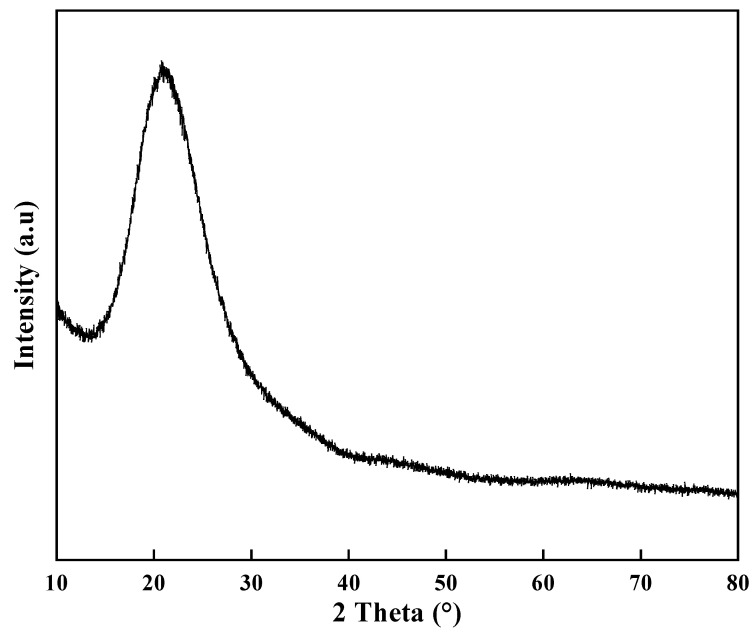
XRD spectrum of microcapsules.

The FTIR spectra of nano-silica, modified n-alkane, and microcapsule were shown in Figure 6. Nano silica was prepared by the hydrolysis and polycondensation of TEOS and APTS. The absorption peaks at 2923 cm^−1^ and 2856 cm^−1^ are considered as C–H stretching vibration of –CH_3_ and –CH_2_, which are also present in nano-silica due to the residue of reactants. Moreover, the absorption peak at 1462 cm^−1^ corresponds to C–H bending vibration of –CH_3_, and the one at 721 cm^−1^ results from the –CH_2_ in-plane rocking vibration. In addition, the appearance of the peak at 1056 cm^−1^ of nano-silica and microcapsule is associated with Si–O–Si stretching vibration. No obvious new peak appears in the microcapsules. It can be seen from the above results that the modified n-alkanes are effectively coated by nano-silica. Figure 7 illustrates the XRD spectrum of microcapsules. A wide dispersion peak appears at 21°, indicating the amorphous structure of the produced nano-silica. Meanwhile, there is no characteristic peak in the spectrum of microcapsules because the modified n-alkanes have no liquid crystal under the test conditions.

### 3.3. Thermal Conductivity and Reliability

**Figure 8:**
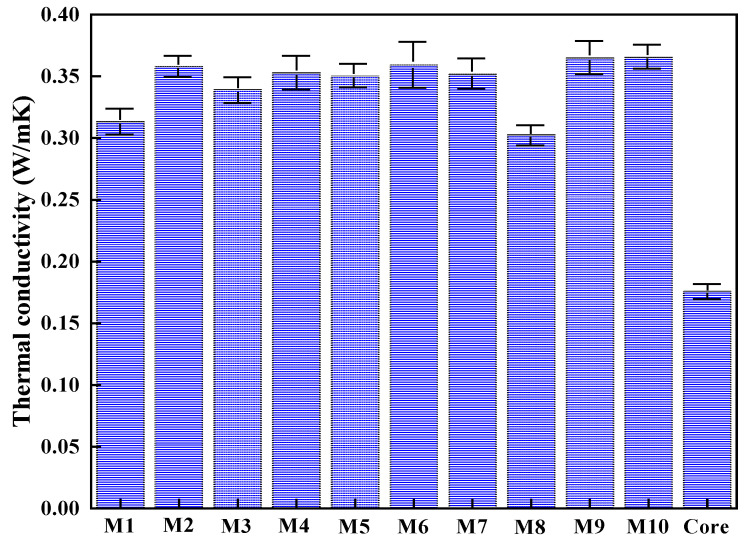
Thermal conductivity of different microcapsules and core material.

As organic PCM, the modified n-alkane has the inherent defect of low thermal conductivity. Figure 8 displays the thermal conductivity of different microcapsules and modified n-alkane. The average thermal conductivity of liquid modified n-alkane is only 0.1755 W/mK, which can be significantly improved by the coating with nano-silica. With the increase of nano-silica content, the thermal conductivity of microcapsules increases.

**Figure 9:**
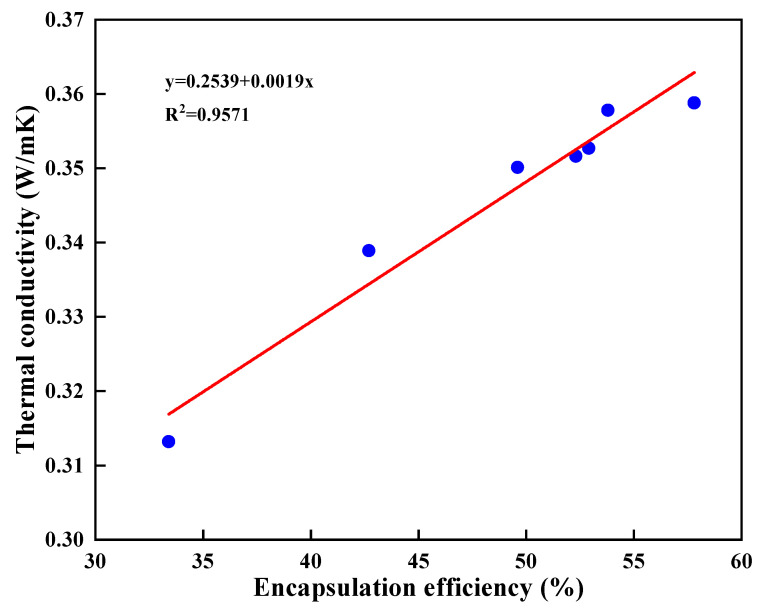
Relationship between encapsulation efficiency and thermal conductivity.

Meanwhile, the relationship between encapsulation efficiency and thermal conductivity under the same dosage of nano-silica was studied in Figure 9, and the results showed a good positive correlation (R^2^ > 0.95). That may be because the higher the encapsulation efficiency, the less the residual voids in the microcapsules, leading to a higher thermal conductivity.

**Figure 10:**
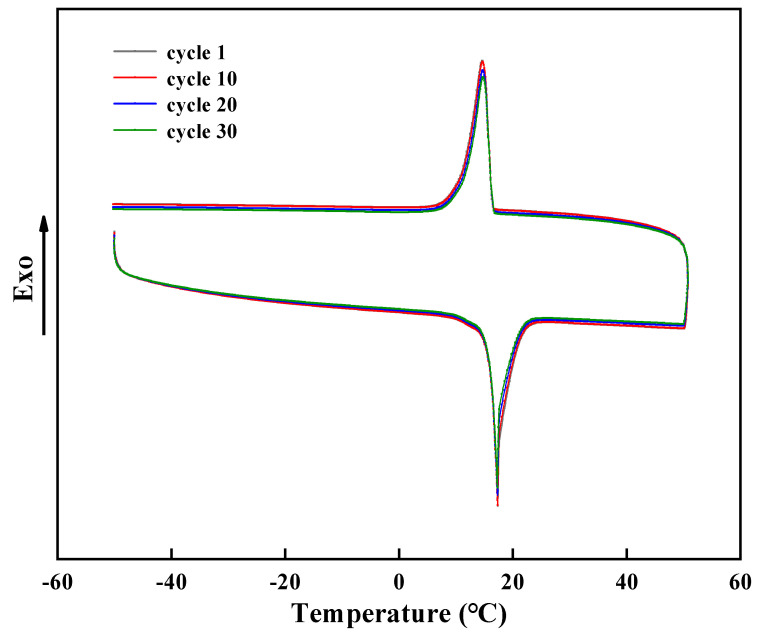
DSC spectra of different thermal cycles.

The thermal reliability of PCMs is the key factor to determine whether they can be recycled [28]. The DSC spectra of microcapsules after different thermal cycles are shown in Figure 10, and it was found that the more the cycle, the smaller the latent heat of microcapsules, indicating that a small number of microcapsules lost efficacy gradually. Compared with the microcapsules after 30 cycles, the melting enthalpy of the initially prepared microcapsules decreased by 9.1%, that is, from 136.8 J/g to 124.3 J/g, which indicates that the microcapsules had relatively good thermal stability.

### 3.4. Thermoregulating Performance and Compatibility with Drilling Fluid

**Figure 11:**
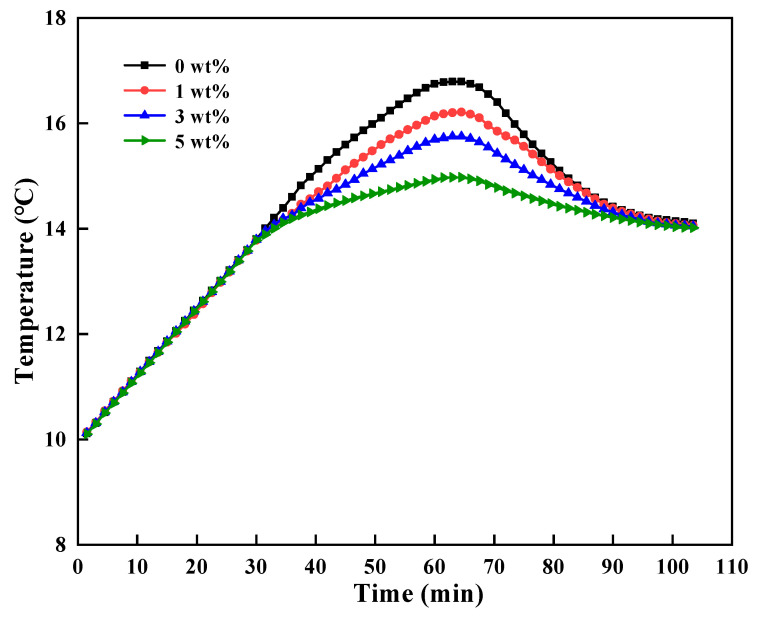
Temperature–time curves of different microcapsule solutions.

From wellhead to seabed, the phase-change microcapsules changed the phase to store energy by cooling and releasing the cold energy in the hydrate formation, thereby controlling the cutting heat released by drilling tools. By simulating the seabed temperature of 3 °C, the cold storage capacity of microcapsules was fully stored; after that, the temperature was increased to 14 °C for the simulation of the temperature of hydrate formation. When the temperature rose to 14 °C, the hotplate stirrer run for 30 minutes to simulate the cutting heat release by the drilling tools. Starting from 10 °C, the solution temperature change was continuously recorded through a high-precision temperature sensor with the temperature-time curves shown in Figure 11. Before reaching the phase transition temperature, the temperature-time curves of different solutions were basically coincident. After that, the solution containing microcapsules showed an obvious trend of temperature rise delay, and with the increase of dosage, the temperature control was more significant. Compared with the pure solution, the maximum temperature of the solution with 5 wt% microcapsules was only 14.9 °C with a decrease of 1.8 °C, which showed good thermoregulating performance of microcapsules.

**Figure 12:**
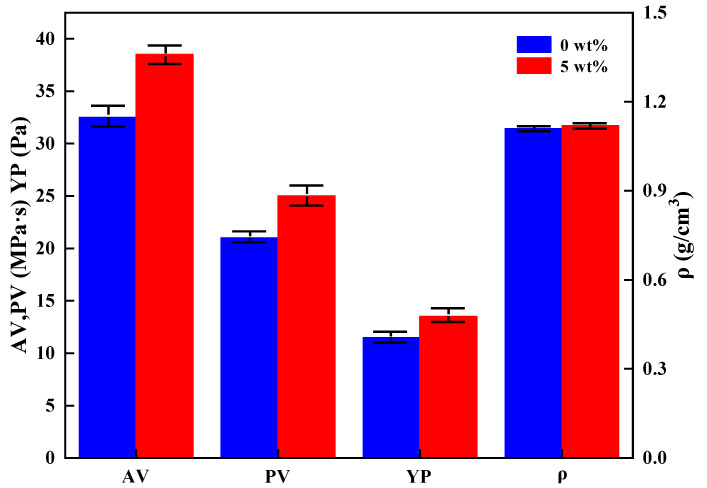
Rheological parameters and density of drilling fluid with or without microcapsules.

**Figure 13:**
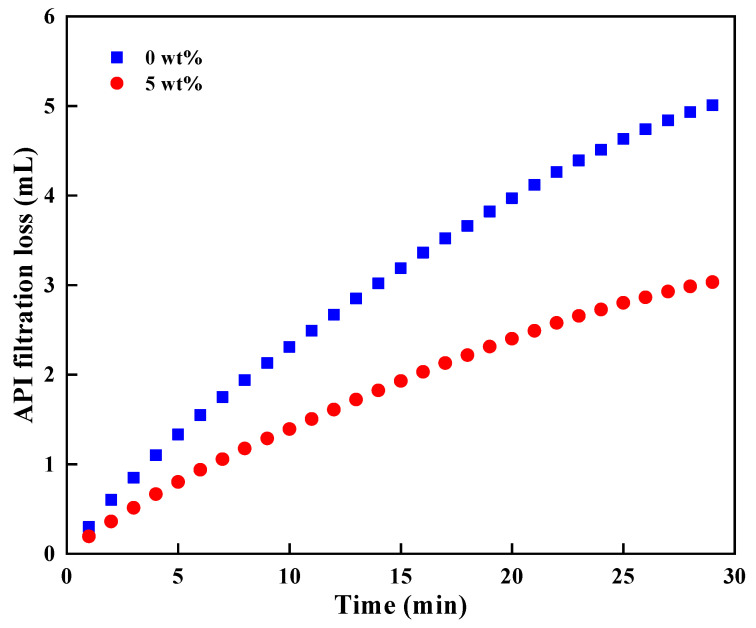
API filtration loss of drilling fluid with or without microcapsules.

Water-based drilling fluid system has been used for hydrate formation drilling [24,25], and the microcapsules should have a good compatibility with drilling fluid in the practical application. Figure 12 and Figure 13 show the basic properties of drilling fluid (previously constructed for drilling in hydrate formation [33]) with and without microcapsules, respectively. It is found from the results that the addition of microcapsules can increase the viscosity of drilling fluid, and the yield point to plastic viscosity remained at 0.5, which is conducive to carrying cuttings and cleaning the bottom hole [34,35]. In addition, the addition of microcapsules had little effect on the density of drilling fluid, and there was no density difference between the upper and lower layers after standing for 72 h, indicating the good solubility and settlement stability of microcapsules in drilling fluid. By comparison, the addition of 5 wt% microcapsules can reduce the filtration loss from 5 mL to 3.1 mL, and it can significantly reduce the impact of filtrate invasion on the original formation, which was of great significance to maintain the wellbore stability of hydrate formation.

## 4. Conclusions

Microcapsules with modified n-alkane as core material and nano-silica formed by simultaneous hydrolysis and polycondensation of TEOS and APTS as shell material were synthesized by interfacial polymerization. The latent heat of the prepared microcapsules was 136.8 J/g in the case of melting enthalpy and 136.4 J/g in the case of solidification enthalpy at the encapsulation efficiency as 62.6%. The microcapsules were in spherical shape, and the median particle size was 10.341 μm, which was conducive to the recycling through the screen. In addition, the coating of nano-silica improved the thermal conductivity of the modified n-alkane. After 30 cycles, the microcapsules still exhibited good thermal reliability. With the addition of 5 wt% microcapsules, the solution showed good thermoregulating performance, which was of great significance for the inhibition of formation hydrate decomposition. Moreover, the microcapsules showed good compatibility with water-based drilling fluid. Therefore, this paper proposed a new idea for the inhibition of formation hydrate decomposition by intelligent temperature control of drilling fluid, and developed a phase-change microcapsule available to be applied to hydrate drilling engineering, which has guiding significance for the design of drilling fluid in hydrate formation.

## Figures and Tables

**Table 1:** Latent heat properties of the microcapsules under different conditions.

Sample	Oil/Water Ratio (*w*/*w*)	Emulsifier Ratio (*w*_Span_/*w*_Tween_)	Core/Shell Ratio (*w*/*w*)	Melting		Solidification		Encapsulation	
				Temp (°C)	Enthalpy (J/g)	Temp (°C)	Enthalpy (J/g)	Rate (%)	Efficiency (%)
M1	10:80	1.5:1.5	10:10	13.94	72.6	13.91	73.0	33.6	33.4
M2	10:100	1.5:1.5	10:10	13.95	117.8	13.94	117.2	54.5	53.8
M3	10:120	1.5:1.5	10:10	13.88	93.4	13.89	93.1	43.2	42.7
M4	10:100	1.3:1.3	10:10	14.00	115.4	13.98	115.8	53.4	52.9
M5	10:100	1.7:1.7	10:10	13.97	108.7	13.96	107.9	50.3	49.6
M6	10:100	1.2:1.5	10:10	13.97	126.1	13.95	126.0	58.4	57.8
M7	10:100	1.0:1.5	10:10	13.92	114.4	13.90	114.1	52.9	52.3
M8	10:100	1.2:1.5	10:8	14.01	84.3	14.00	81.6	39.0	38.0
M9	10:100	1.2:1.5	10:12	13.99	136.8	13.95	136.4	63.3	62.6
M10	10:100	1.2:1.5	10:14	13.89	123.6	13.88	121.6	57.2	56.2

**Table 2:** The comparison of encapsulation efficiency of different PCMs.

Sample	Melting Enthalpy (J/g)	Encapsulation Efficiency (%)	Ref.
Polystyrene /n-tetradecane	98.7	44.3	[19]
MF /n-tetradecane	100.3	44.2	[20]
polyHIPE /n-hexadecane	143.4	56.3	[31]
CaCO_3_ /n-tetradecane	58.5	25.9	[32]
Silica /modified n-alkanes	136.8 (±2.0)	62.6 (±0.8)	This study
